# Relative instability ratios of bone wall defects in trochanteric hip fractures: A finite element analysis

**DOI:** 10.3389/fbioe.2023.1082613

**Published:** 2023-02-03

**Authors:** Ao-Lei Yang, Wei Mao, Shi-Min Chang, You-Hai Dong

**Affiliations:** ^1^ Department of Orthopedic Surgery, Yangpu Hospital, Tongji University School of Medicine, Shanghai, China; ^2^ Department of Orthopaedics, Fifth People’s Hospital of Shanghai, Fudan University, Shanghai, China

**Keywords:** medial wall, lateral wall, relative instability ratio, finite element analysis, trochanteric hip fracture

## Abstract

**Background:** For decades, medial and lateral wall fragments of trochanteric hip fractures were considered two pivotal factors that could influence the stability of postoperative femur-implant complex. However, most studies seemed to misunderstand the concept of the posteromedial fragment and equated it with the medial wall, which overlooked vital roles of the anteromedial wall. Roles of the posterior coronal bone fragment were also highlighted in some research. However, influences of the bone walls above the trochanteric fracture instability are yet to be investigated and quantified by means of finite element analysis.

**Methods:** Eight trochanteric fracture fixation models with different wall defects were constructed. Outcome indicators were the von Mises stress of the implant models, the maximum/minimum principal strain, the risky tensile/compressive volume and the volume ratios of the bone models, the femoral head vertex displacement, and the fracture surface gap. Based on these indicator values, the relative instability ratios were computed.

**Results:** Outcome indicators, absolute values, and nephograms of all models showed the same upward and concentrating trends with exerted hip contact loads shifting from static walking to dynamic climbing. Similarly, these indicators also exhibited the same trends when the eight models were solved in sequence. Moreover, the relative instability ratio of the medial wall (100%), particularly the anteromedial part (78.7%), was higher than the figure for the lateral wall (36.6%).

**Conclusion:** The anteromedial wall played relatively pivotal stabilizing roles in trochanteric hip fractures compared with the posteromedial wall and the lateral wall, which indicated that orthopedic surgeons should attach more importance to the anteromedial cortex support in an operating theatre.

## 1 Introduction

Trochanteric hip fractures, accounting for a significant proportion of hip fractures, are not always caused by a single fracture line occurring within the intertrochanteric zone defined by the latest 2018 revision of the Arbeitsgemeinschaft fur Osteosynthesefragen (AO/OTA) classification system (Bhandari and Swiontkowski, 2017; Meinberg et al., 2018). On the contrary, there are clinically far more fracture lines than single ones that spilt up the intertrochanteric region into more fragments, which more often than not augment the instability of the trochanteric fractures (Zhang et al., 2020). Moreover, up to 50%–60% of the trochanteric fractures are classified as unstable (Wu et al., 2019), and the condition will directly determine surgical treatments and intramedullary or extramedullary fixation. According to a consensus reached by recent research and proposed as the guideline by the AO/OTA classification (2018), intramedullary fixation systems [e.g., the proximal femoral nail anti-rotation (PFNA, Depuy Synthes, United States), the Gamma nail 3 (Stryker, Mahwah, New Jersey), and the intertrochanteric antegrade nail (InterTAN, Smith & Nephew, Memphis, Tennessee)] are recommended as primary protocols for unstable fractures to minimize potential complications (Müller et al., 1990; Zhang et al., 2013; Meinberg et al., 2018; Fu et al., 2020; Lee et al., 2020; Yapici et al., 2020).

With the rapid development of and innovation for surgical instruments, ideas, procedures, and patient-centered care, surgeons can now rigorously formulate individual treatment and try their utmost to diminish unstable elements for better prognoses of their patients. Simultaneously, that mandates orthopedic clinicians ascertain every subtle risk factor initially and pay more attention to them over the whole therapeutic course. Obviously, according to the 1990 and 2018 guidelines in the AO/OTA classifications, two vital factors have been designated: the posteromedial fragment with its extension downwards and the lateral wall fragment with its thickness < 20.5 mm (Müller et al., 1990; Meinberg et al., 2018). However, none of these were surgically highlighted nor fixed in many trochanteric fractures with the use of intramedullary fixation (Liu et al., 2015; Puram et al., 2017; Wu and Tang, 2019; Chang et al., 2022; Yang et al., 2022), from which some researchers concluded that the medial walls were of minor importance in the fracture stability.

But there were also some studies arguing that it was a misunderstanding to simply equate the posteromedial wall and the medial wall. They also demonstrated that it was the anteromedial wall that played a pivotal function and hence anatomically divided the medial wall into the anteromedial wall and the posteromedial wall (Chang et al., 2018; Chen et al., 2020; Zhang et al., 2020; Shao et al., 2021). Moreover, roles of the posterior coronal fragments have also attracted more investigations recently. However, controversy arose about which were the more significant unstable elements between the medial wall and the lateral wall and between the anteromedial wall and the posteromedial wall with or without intramedullary fixation (Müller et al., 1990; Nie et al., 2017; Chang et al., 2018; Meinberg et al., 2018; Chang et al., 2020; Chen et al., 2020).

Although the AO/OTA (2018) highlighted the lateral wall thickness and put it as a standard to evaluate whether a trochanteric fracture was stable, some researchers remained skeptical about the significance level of the lateral wall (Nie et al., 2017; Wu and Tang, 2019; Chang et al., 2022). With the prevalence of the intramedullary fixation used for unstable trochanteric fractures, which was also recommended by the AO/OTA (2018), Chang et al. (2022) put forward a conception of a “metal lateral wall”. This meant the intramedullary nail could take over most roles of the actual lateral wall, even if the lateral wall broke during or after the operation. Moreover, some researchers still further demonstrated that it was the anteromedial wall and not the posteromedial wall that played the pivotal stabilizing role (Nie et al., 2017; Chen et al., 2020; Zhang et al., 2020).

Therefore, in this study, influences of the bone walls on trochanteric fracture instability were explored and quantified with contribution ratios by means of the finite element analysis.

## 2 Methods and materials

Two patients’ (a 73-year-old man: 174 cm, 70 kg, 23.12 kg/m^2^ and a 72-year-old woman: 170 cm, 70 kg, 24.22 kg/m^2^) historical computed tomography (CT) images (slice thickness, 1.0 mm) of bilateral femurs were gathered from a data archive of the Fifth People’s Hospital of Shanghai. Both patients had the same dual-energy x-ray absorptiometry (DEXA) test value with T-Score = −2.0 and were in a healthy state without fracture histories.

### 2.1 The fracture models

The gathered data of CT images were input to Mimics Research 21.0 (Materialise N.V., Leuven, Belgium) to construct three-dimensional femur models, including two left femur models and two right femur models. Next, the constructed entire femur models were imported into 3-Matic Research 13.0 (Materialise N.V., Leuven, Belgium) to further establish trochanteric fracture models.

**FIGURE 1:**
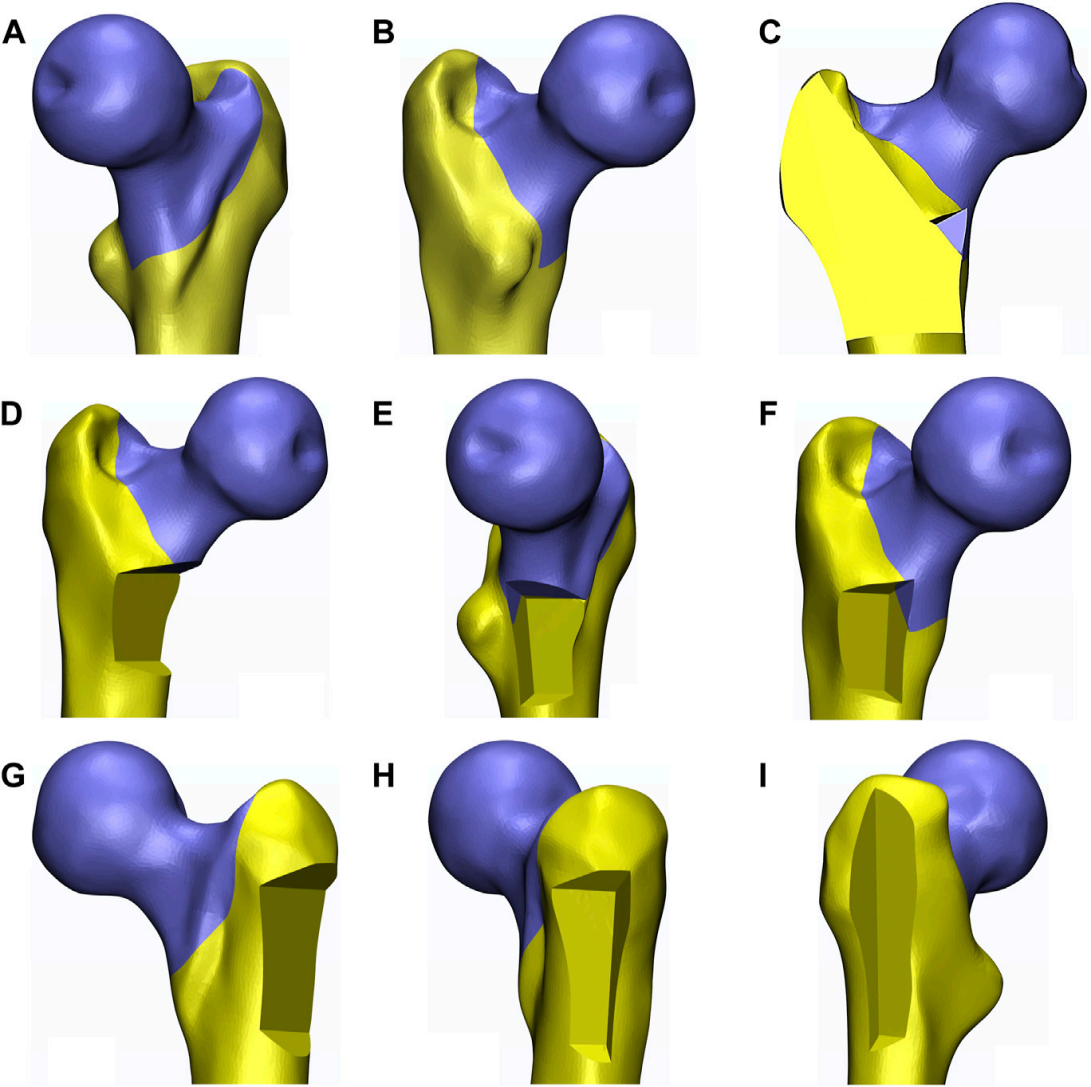
The diagram exhibited the constructed eight fracture models based on one left femur (also the following figures). **(A,B)** indicated different perspectives of the basic fracture model. **(C–I)** indicated the posterior coronal bone defect model, the medial, the anteromedial, the posteromedial, the lateral, the anterolateral, and the posterolateral wall defect model, respectively.

With this software, the corresponding eight distinct models were constructed according to the three-dimensional mapping of trochanteric fracture lines, as shown in Figures 1A, B (Li et al., 2019; Zhang et al., 2020). The basic fracture line was set as an irregular curve. The lateral wall area and the corresponding defect were also referred to the definition of Haq et al. (2014), while the medial wall area and the corresponding defect were set as the region within 2 cm above and below the lesser trochanter, which was an important zone when testing stress with its deep dense calcar femoral (Kuzyk et al., 2012; Chang et al., 2020; Lee et al., 2020). Moreover, based on the fundamental fracture model, the defect depth of both the medial wall and the lateral wall were set as half of the distance between their respective cortex to the femoral shaft axis. Then the medial wall and the lateral wall were cut by the projection line of the femoral shaft axis into the anterior medial (AM) wall and the posterior medial (PM) wall, and the anterior lateral (AL) wall and the original posterior lateral (PL) wall, respectively, as displayed in Figure 1D–H. However, the upper region of the original PL wall defect model was more involved in realistic trochanteric fracture fragments (Li et al., 2019). Therefore, the region was also removed to establish the current PL wall defect model, as shown in Figure 1I. In addition, the posterior coronal bone (PML) defect model was constructed by cutting the PM wall and the PL wall as well as the rest of the posterior bone between them, as exhibited in Figure 1C.

### 2.2 The PFNA models

**FIGURE 2:**
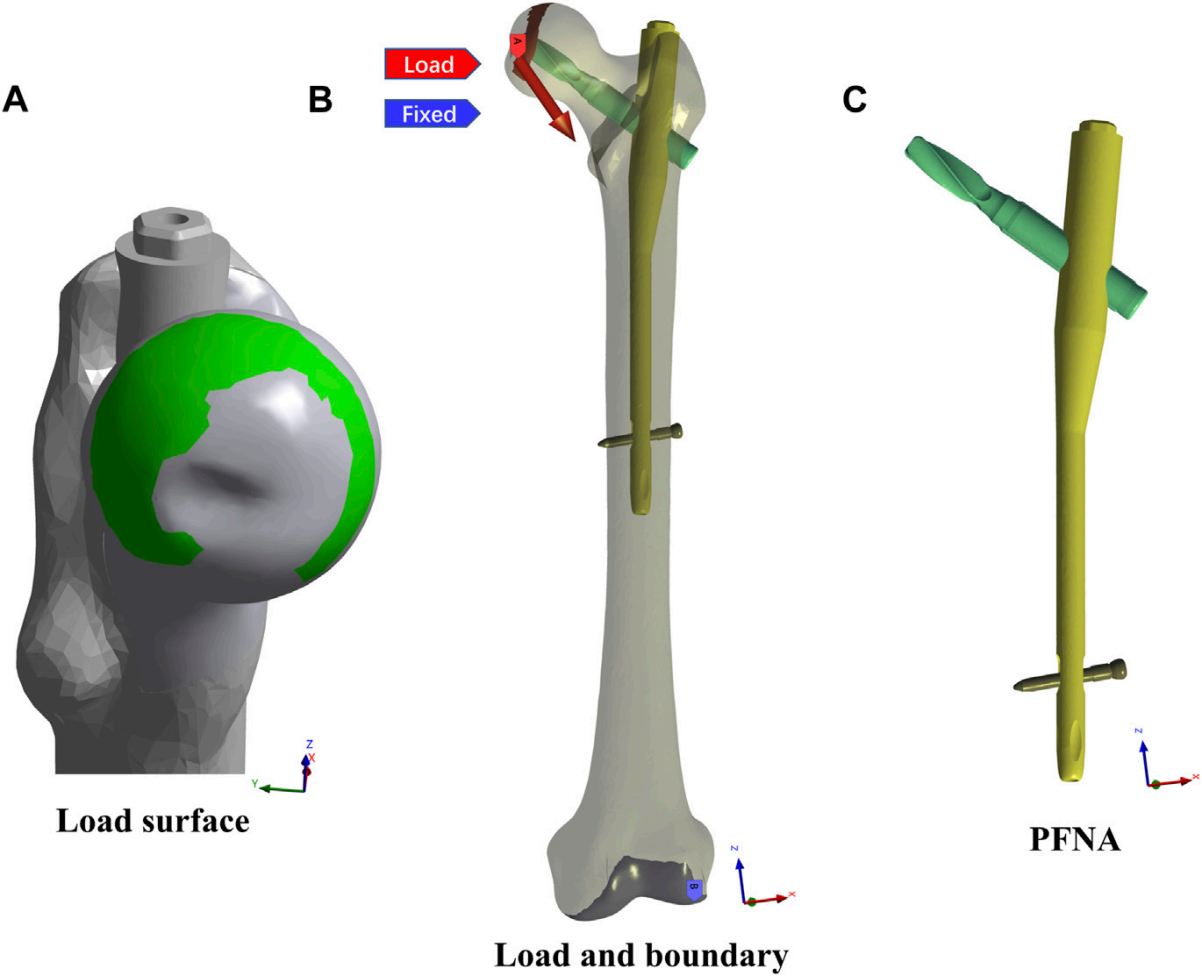
The load, boundary conditions, and the PFNA model were exhibited. **(A)** showed the load surface, on which the static walking and climbing hip loads were exerted. **(B)** showed the static load site and direction and the FE model fixed site. **(C)** showed the PFNA model.

Dimensions of an intramedullary fixation kit of titanium alloy, PFNA (length: 200 mm and an angle: 125°), including an intramedullary (IM) nail, a helical blade, and a locking screw, were obtained from the manufacturer (Depuy Synthes, United States), as shown in Figure 2C. Then the primary file was also input to 3-Matic software to reach the element congruity of the implants with the counterpart of the fracture models before their final assembly.

### 2.3 The fracture fixation and finite element models

With 3-Matic software, the revised PFNA model and each fracture model were then implemented into the assembly in accordance with clinical standards. To maintain the intra-group consistency of the implant positions, in each model the IM nail was identically located in the intermediate region inside the femoral canal while the helical blade was anchored in the intermediate-inferior region within the femoral head. This was a critical procedure that ensured that the tip-apex distance (TAD) of each model was 15 mm. After the establishment of every fracture fixation model, holistic volume elements were remeshed and modified; the final mesh quality check of all models met the software-provided criterion about the homogeneous property of all volume elements of each model.

For each model, the material assignment was exercised in Mimics software, in which mapping approaches of CT-Hounsfield unites and grey values were used to convert the matched voxels into the bone density (*ρ*) and the elastic modulus (E) of the corresponding volume mesh (Morgan et al., 2003; Taddei et al., 2007). Formulae in the guidebook of Mimics about the bone material assignments were as follows: *ρ* = 131 + 1.067 HU (kg/m^3^); E = −331 + 4.56 *ρ* (MPa). Moreover, the Poisson’s ratio of the bone was set as 0.3, and the Poisson’s ratio and the elastic modulus of the implants of titanium alloy were set as 0.35 and 105,000 MPa, respectively (Goffin et al., 2013).

Each material-assigned model in 3-Matic software was then discretized into ten-node tetrahedral elements of the solid-185 volume. All materials were presumed to be homogeneous, linearly elastic, and isotropic (Hawks et al., 2013). Using standard deviation caused from repeating all steps, including manual adjustments of the surface/volume mesh, of each FE model three times, mean element numbers of the implant model and the eight fracture fixation models were 46,094 ± 805, 167,430 ± 3,104 (the basic fracture), 143,340 ± 2,340 (the M wall defect), 143,986 ± 2,315 (the AM wall defect), 145,434 ± 2,214 (the PM), 145,286 ± 2,615 (the L), 150,920 ± 2,235 (the AL), 146,404 ± 2,803 (the PL), and 143,452 ± 2,185 (the PML).

Next, all disposed finite element (FE) models were imported to ANASYS Workbench (ANSYS Inc., version 2021 R1) to proceed with further simulation.

### 2.4 Friction, loading, and boundary conditions

**FIGURE 3:**
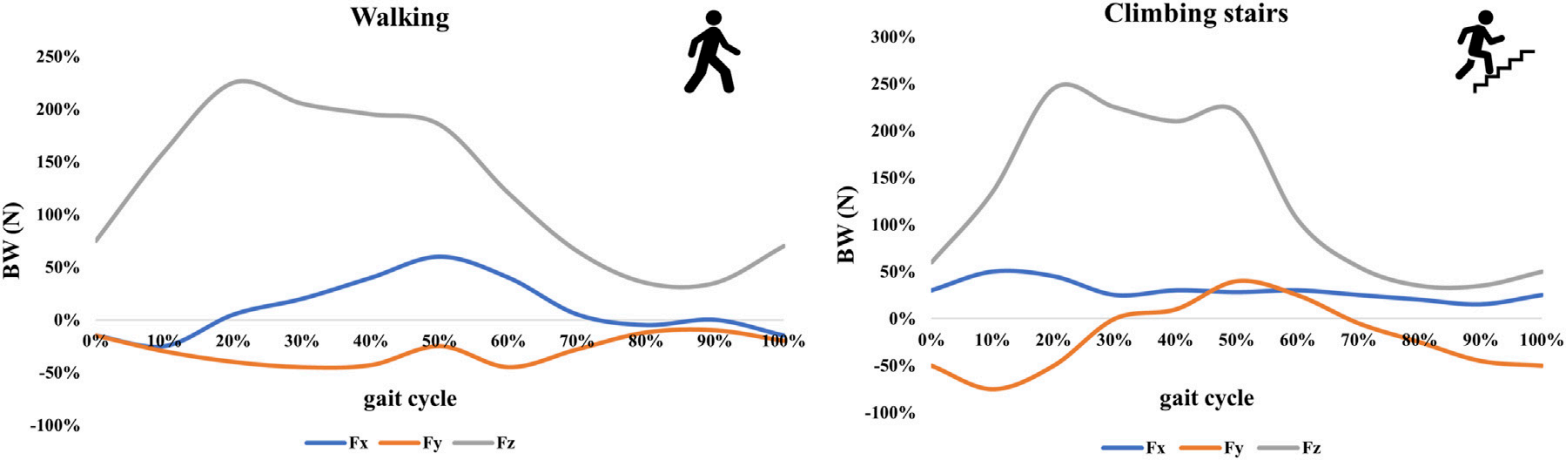
The load curves of dynamic walking and climbing stairs cycle gaits.

Contact interaction between every part of each FE model were set to be frictional in order to approach the reality, and the surface friction coefficients between the bone-bone, the bone-implant, and the implant-implant were set as 0.46, 0.42, and 0.2, respectively (Hsu et al., 2007; Eberle et al., 2010; Lee et al., 2016). The loading conditions were adopted with two kinds of common cycle gaits, namely walking and climbing stairs, and under each of them, static and dynamic hip contact loads were simulated and exerted on the head load contact surfaces of all FE models, as exhibited in Figure 2A. The static hip contact loads in normal walking and climbing cycle gaits were set as follows as to a person weighing 70 kg (Heller et al., 2005; Taheri et al., 2011; Ramos et al., 2016; Cun et al., 2020): the hip joint contact load in the walking gait [(x,y,z) = (647, 236, −1,351)] and in the climbing gait [(x,y,z) = (711, 436, −1,393)], which were 2 times and 2.1 times body weight, respectively. Meanwhile, the dynamic hip contact loads in the two cycle gaits were adopted with curves, as shown in Figure 3 (Chalernpon et al., 2015; Zeng et al., 2020; Jiang et al., 2022). Furthermore, the distal end of the FE model was fully constrained both in translation and rotation as the boundary condition in all models, as displayed in Figure 2B (Heller et al., 2001; Heller et al., 2005).

Simultaneously, the mesh convergence was tested in ANSYS to evaluate the validity and veracity of each FE model. Additionally, each model had been constructed and simulated three times in order to reduce errors as much as possible. After the solution of each model, outcome indicators were set as follows: von Mises stress (VMS) of the implant models, including the IM nail and the helical blade; maximum/minimum principal strain, tensile/compressive volume, and volume ratios of the bone models; and the displacement distance of the FE model head vertex toward the distal femur and the fracture surface gap of each FE model. These were then determined and analyzed.

### 2.5 The relative instability ratios

Owing to little previous study or guidelines on how to measure the stability of the trochanteric fracture-implant complex, a novel concept, namely relative instability, was put forward to indirectly assess the significance of each trochanteric fracture wall defect. However, a substantial amount of research concluded that, although no linear relation between these indicator absolute values mentioned above and instability of the corresponding models was inferred, the FE model indeed went toward a yielding failure with the local indicator values gradually increasing (Goffin et al., 2013; Lee et al., 2016; Nie et al., 2020; Lewis et al., 2021; Mohammad et al., 2022). Therefore, for each indicator outcome above all FE models, the highest absolute value was supposed to be relatively the most unstable with a relative instability ratio of 100%, while its opposite pole was relatively stable with 0%. Then the relative instability ratios of the other models were calculated as to where their values stood from the lowest value to the highest value. In the last step, these ratios of each model were statistically analyzed.

## 3 Results

The outcome indicators of each FE model all showed the same increasing trends in absolute values and/or a gradually concentrating tendency in nephograms with the exerted hip contact loads shifting from the static walking gait to the dynamic climbing stairs gait. Similarly, almost all these indicators also exhibited the same upsending tendencies when the FE models were solved in sequence from the basic fracture line model, the PL wall defect model, the AL, the PM, the L, the PML, the AM, to the M wall defect model.

### 3.1 The von Mises stress of the implant models

**FIGURE 4:**
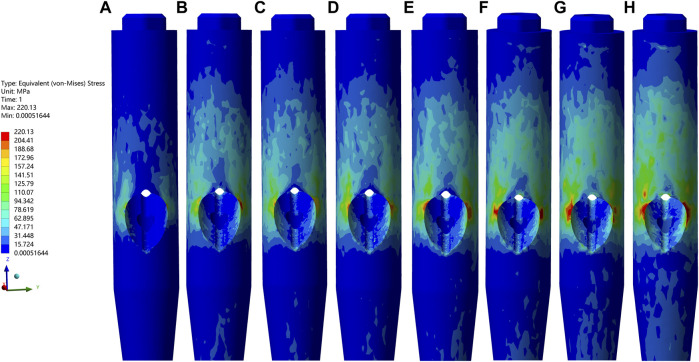
The VMS values and nephograms of the IM nails under the dynamic climbing hip load were exhibited. **(A–H)** indicated the basic fracture line model, the PL wall defect model, the AL, the PM, the L, the PML, the AM, and the M wall defect model, respectively. From images **(A–H)**, the peak value of each IM model gradually increased and the highest one of 220.13 MPa appeared in **(H)**.

**FIGURE 5:**
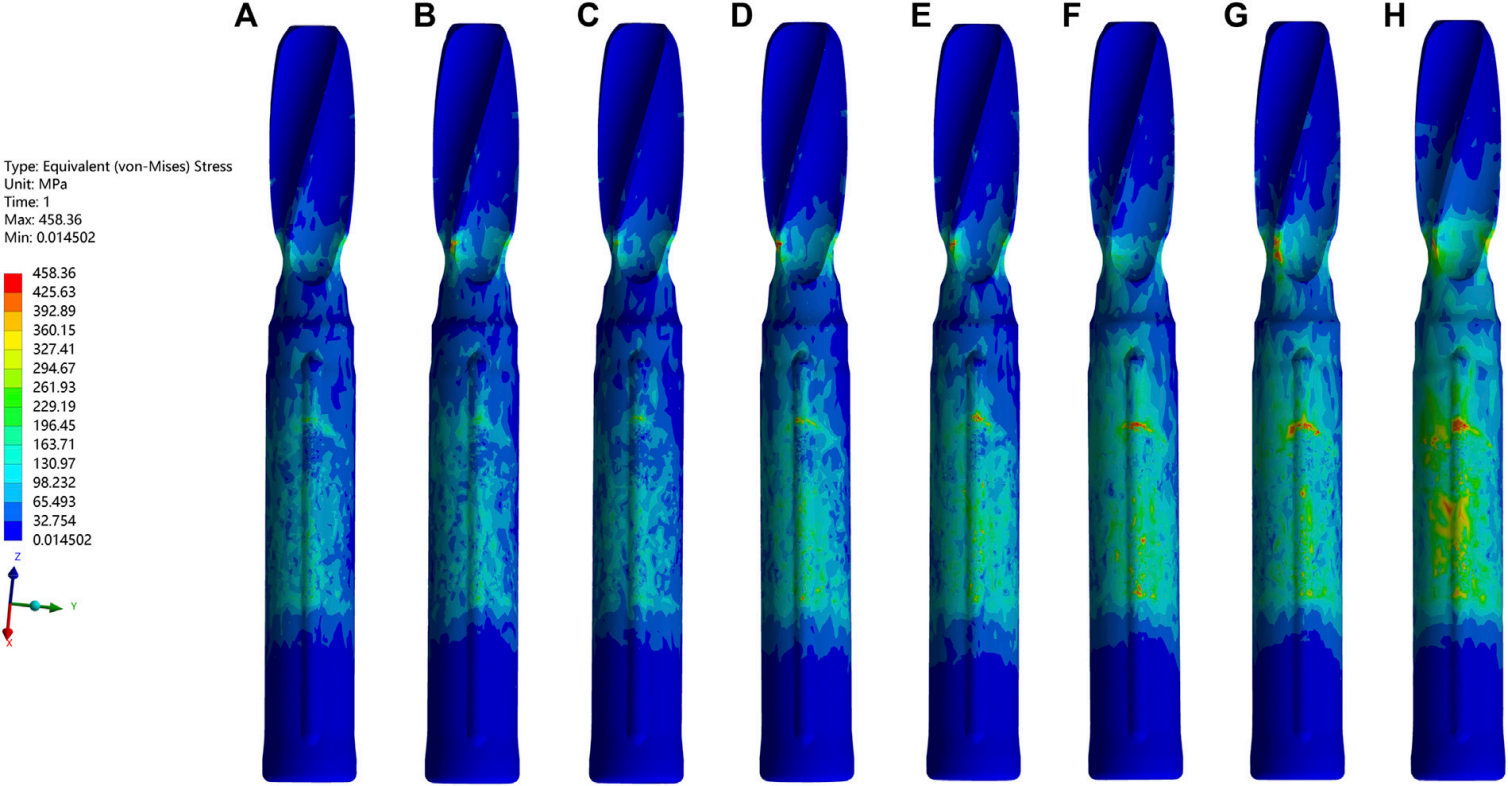
The VMS values and nephograms of the blades under the dynamic climbing hip load were exhibited. The order was the same as it was in Figure 4. From images **(A–H)**, the peak value of each blade model gradually increased and the highest one of 458.36 MPa appeared in **(H)**.

**FIGURE 6:**
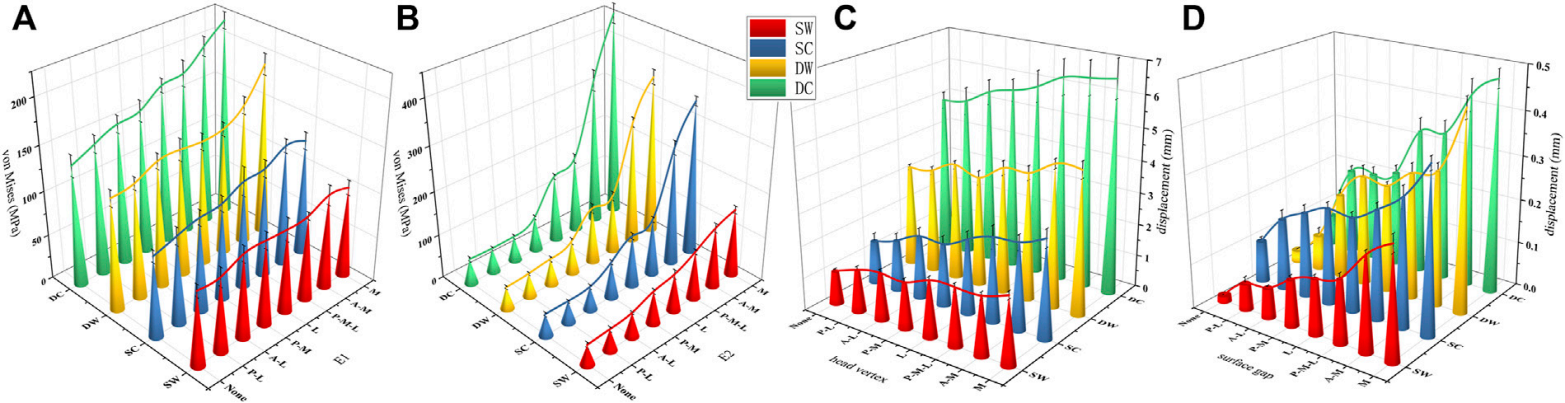
The von Mises stress curves of the IM nails (E1) and the helical blades (E2) were exhibited in **(A,B)**, respectively. The head vertex displacement and the surface gaps were exhibited in **(C,D)**, respectively. SW, SC, DW, and DC indicated the static walking, the static climbing, the dynamic walking, and the dynamic climbing, respectively. None indicated the basic fracture model while the others indicated the same as mentioned above.

In all cases, the peak VMS values and the nephogram concentrating sites of the IM nail models and the helical blade models showed a common area at the conjunction of each pair of the IM nail and the blade, especially the superior spot of the lateral hole of the IM nail model and the blade model spot contacting the inferior spot of the medial hole of the corresponding IM nail model. Furthermore, the peak VMS value, 220.13 MPa, of all IM nail models occurred in the medial wall defect model under the dynamic climbing hip loads. Simultaneously, the peak value of 458.36 MPa of all helical blade models was also generated in the same model, as shown in Figures 4–6. However, none of these peak values exceeded the yielding stress of 750–900 MPa for the implants of titanium material (Sitthiseripratip et al., 2003; Levadnyi et al., 2017).

### 3.2 The maximum and minimum principal strain of the bone models

**FIGURE 7:**
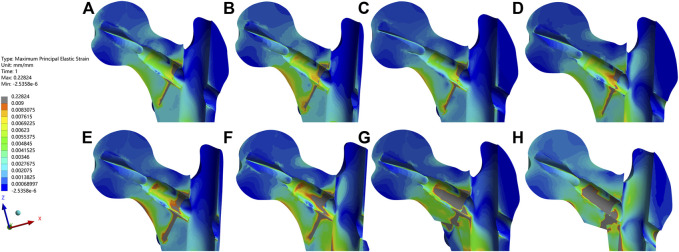
The maximum principal strain and nephograms of the proximal fracture models under the dynamic climbing hip contact loads were exhibited. The order was the same as it was in Figure 4. From images **(A–H)**, the peak value of each bone model gradually increased and the highest one of 0.22824 appeared in **(H)**. The elements with the values above the cut-off of 0.009 were marked in dark gray.

**FIGURE 8:**
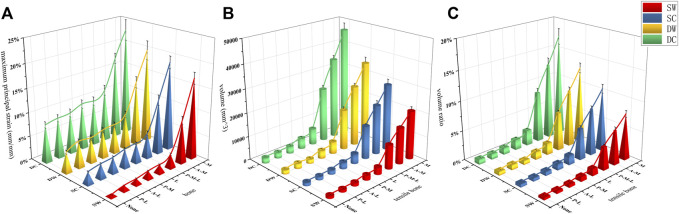
The maximum principal strain, the risky tensile bone volume, and the volume ratio curves of the bone models were exhibited in **(A–C)**, respectively.

**FIGURE 9:**
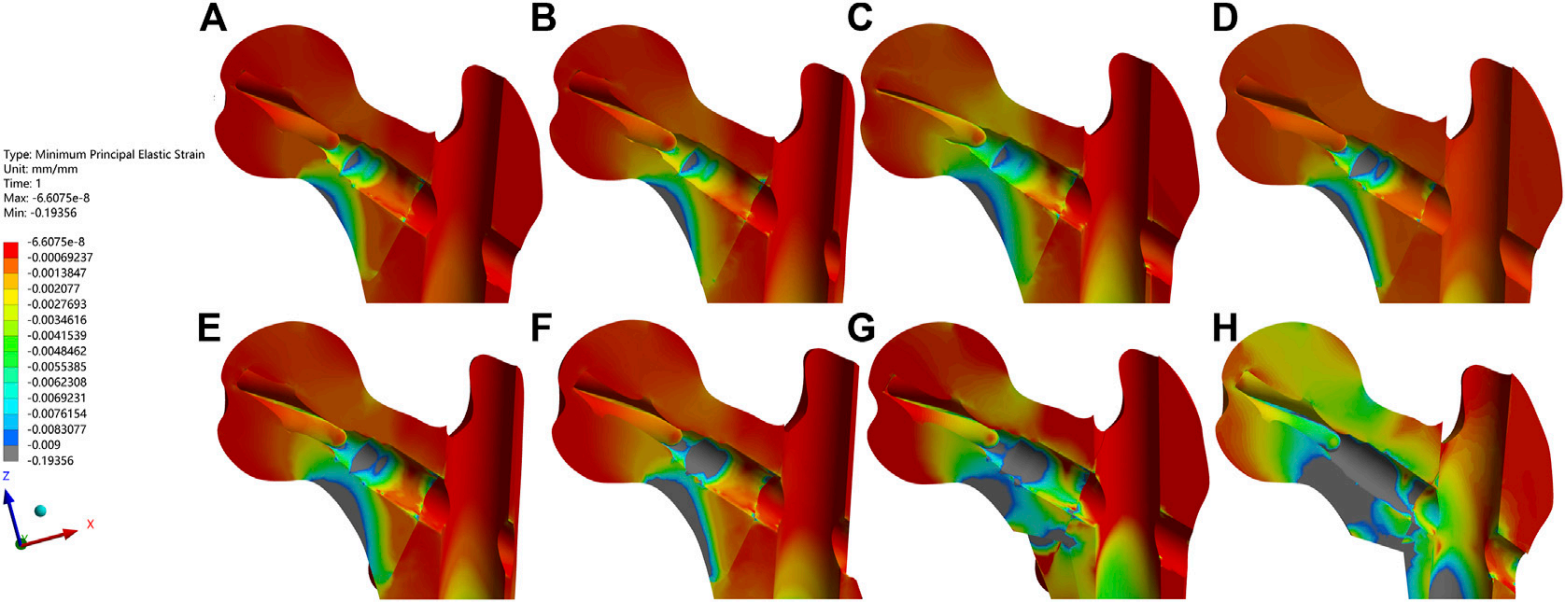
The minimum principal strain and nephograms of the proximal fracture models under the dynamic climbing hip contact loads were exhibited. The order was the same as it was in Figure 4. From images **(A–H)**, the least value of each bone model gradually decreased and the lowest one of −0.19356 appeared in **(H)**. The elements with the values below the cut-off of −0.009 were marked in dark gray.

**FIGURE 10:**
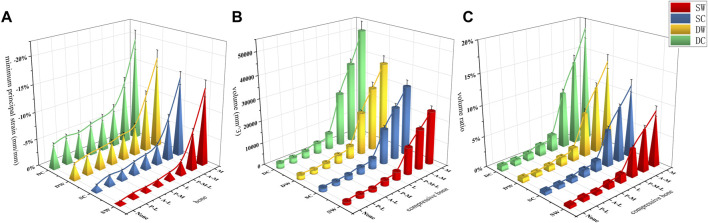
The minimum principal strain, the risky compressive bone volume, and the volume ratio curves of the bone models were exhibited in **(A–C)**, respectively.

The femur bone model elements with the peak maximum (tensile) principal strain above 0.9% and/or the minimum (compressive) principal strain below −0.9% are often interpreted as high-risk locations for the fracture yielding (irreversible deformation) and failure (Kopperdahl and Keaveny, 1998; Panyasantisuk et al., 2016). In addition to the maximum/minimum principal strain, the volumes and the volume ratios of the risky regions were also calculated and analyzed. In the current study, values of the peak maximum principal strain, the risky tensile bone volume, the volume ratio and the peak minimum principal strain, the risky compressive bone volume, and the volume ratio occurred in the medial wall defect model under the dynamic climbing hip loads, which were 22.8%, 46,084 mm^3^, 17.3% and −19.4%, 50,700 mm^3^, and 18.8%, respectively. Furthermore, the risky yielding regions were marked as dark grey in the strain distribution nephograms, and the corresponding value trends were exhibited in Figures 7–10. From the facet of the strain distribution nephograms, the risky tensile bone more likely appeared at the fracture surface with extension toward the blade anchorage region while the risky compressive bone was more likely at the blade anchorage region with extension toward the medial and inferior femoral cortex.

### 3.3 The femoral head vertex displacement and the fracture surface gap of the FE models

The femoral head vertex displacement of the FE models toward the distal femur (z-axis) ranged from 4.91 ± 0.25 to 6.59 ± 0.59 mm, while the fracture surface gaps ranged from 0.073 ± 0.006 to 0.478 ± 0.02 mm. The mean values and trends of these indicators were shown in Figures 6C, D.

### 3.4 Relative instability ratios of bone wall defect models

**FIGURE 11:**
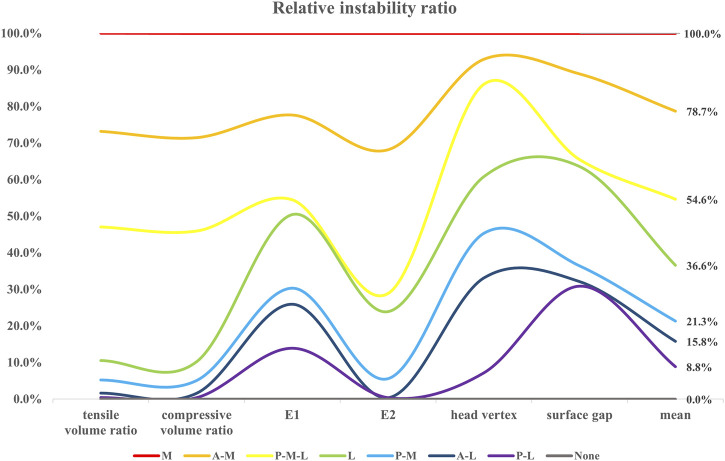
The relative instability ratio of each model concerning the six outcome indicators were exhibited, and the corresponding mean values were shown in the last row.

Owing to the stability of proximal trochanteric fractures being largely contingent on fragments of the femoral head-neck, the femoral shaft and implants, including the IM nail, and the helical blade, the outcome indicators above were all taken into consideration to evaluate the relative instability ratio of each bone wall defect model. According to the trends of all indicators, the relative instability of the basic fracture models was presupposed to be 0%, while the medial wall defect model was presupposed to be 100%. Then the relative instability order and ratios, which were exhibited in Figure 11, were as follows: the basic fracture line model (0%), the PL wall defect model (8.8%), the AL (15.8%), the PM (21.3%), the L (36.6%), the PML (54.6%), the AM (78.7%), and the M wall defect model (100%).

## 4 Discussion

According to the three-dimensional mapping of trochanteric fractures lines (Zhang et al., 2020), eight trochanteric fracture models were constructed and simulated in the current study. Contrary to most previous relevant finite elements analysis research, the current study adopted a more realistic irregular curve fracture line and both static and dynamic hip contact loads, which were further divided into walking and climbing gait cycles. The selection of outcome indicators referred to a compelling biomechanics review (Lewis et al., 2021), which suggested that von Mises stress was more appropriate and widely utilized to evaluate the stability of the implant models, while the maximum/minimum principal strain should be used to assess the bone models. Therefore, to take more predictive factors into consideration about the FE model instability, the von Mises stress (i.e., equivalent stress) of each implant model, the maximum/minimum principal strain, tensile/compressive volume and volume ratios of each bone model, the displacement of the femoral head vertex, and the fracture surface gap were calculated and analyzed.

After the holistic analysis, the absolute values of all indicators above presented nearly the same trends. When it came to a single model, all absolute values exhibited upward tendencies when the hip contact loads shifted from static walking, to static climbing, to dynamic walking, to dynamic climbing. As for all eight models, all absolute values also manifested increasing trends when the solution of each model shifted from the basic fracture model, the PL wall defect model, the AL, the PM, the L, the PML, and the AM, to the M wall defect model. What’s more, the peak values frequently showed in the medial wall defect model under the dynamic climbing hip contact load. That is to say, it was explicit that, when compared with the lateral wall, the medial wall played a far more significant stabilizing role. This was also consistent with conclusions of the previous studies that the role of the lateral wall changed extremely to becoming less important after the IM fixation was fully utilized (Zhang et al., 2020; Shao et al., 2021). As for the medial wall, compared with the posteromedial wall, the anteromedial wall played a predominantly stabilizing role, which was also a biomechanical demonstration for the theory of the anteromedial cortex support reduction (Chang et al., 2020; Chen et al., 2020). It indicated that, with the prevalence of the intramedullary fixation, orthopedic surgeons should prioritize the anteromedial wall conditions of trochanteric fractures to begin with and endeavor to restore their cortex support to gain the most stability out of the fracture-fixation structures in an operating theatre.

In addition, the maximum/minimum principal strain nephograms directly showed the increasingly risky yielding regions around the fracture surfaces and the bone sites contacting the blades, which could also be elucidated with the unique force transmission mechanism within the trochanteric fracture fixation structure. During the initial post-operative period before the fracture healing, with the continuous dynamic trade-off of loading and unloading between the bone and the implant, peripheral loads from the hip would be conducted simultaneously through the cortex and the bone-blade-IM nail. Once the cortex discontinued, stress would surge at the sites around the medial cortex defect and eventually cause the sites to yield and even cause secondary fractures. However, with the “metal lateral wall” role of the IM nail, the discontinued lateral cortex site might suffer from little stress soar, which further had few influences on the fracture-fixation construct stability.

As there is no definite consensus on or measurement methods of “instability of the fracture fixation construct,” the relative instability ratio was adopted in the current study to assess the significance priority of each clinical common defect trochanteric wall. The main unstable elements were set as the outcome indicators above, and the relative ratios of the different wall defect models contributing to the instability of the constructs were set as percentages, with the relatively most stable one occurring in the basic fracture line model (0%) and the relatively most unstable one occurring in the medial wall defect model (100%). Noticeably, all relative ratios computed through the outcome indicator absolute values of the rest of the FE models showed the same position in the sequence order, as exhibited in Figure 11. This finding, to some extent, supported the efficacy of the “relative instability ratio” approach, which was put forward in this study, to evaluate the significance level of a fracture model, an implant model, or the assembly model of them. The approach might then facilitate further studies to make a holistic assessment of their designed unique models with more convincing conclusion. Interestingly, the ratios of the AM wall defect model (78.7%) and the PM wall defect model (21.3%) amounted to the ratio of the M wall defect model (100%), which did not occur in the group of the L wall defect model, the AL, nor the PL. We believe this is due to the “metal lateral wall” role of the IM nail which shared the stability and also the ratio.

There were some strengths of the current study. Firstly, to the best of our knowledge, this was the first study that aimed to investigate contribution ratios of the distinct defect walls to trochanteric fracture instability. Secondly, the material assignment methods of combining CT-Hounsfield and gray value allowed every model in the current study to be exactly simulated with the realistic bone mineral quality. Thirdly, an irregular curve fracture line and the dynamic hip contact loads rendered the FE models closer to the real world with more convincing conclusions. However, some limitations of this study should also be mentioned. On the one hand, the assumption that properties of the material were isotropic, homogeneous, and elastic linearly was just a simplification of the reality. The boundary and constraint of all models were quite simplified compared with that of the actual delicate knee joint, while the exerted loads from hip contact resulted in a dent in actual effects of hip muscles. On the other hand, the adjacent structures and soft tissues, as well as subtle interactions between them and femurs, were ignored in the current study, which might lead to slight differences. Finally, because of the intrinsic limitations of FEA-related software in simulating changes of the bone microarchitecture, such as the cancellous bone collapse, and thermal tensions, and deformability restrictions on each element unit, further cadaveric experiments should be performed to draw more accurate conclusion.

## 5 Conclusion

In the current study, it was demonstrated that the medial wall played a more vital role in trochanteric hip fracture stability than the lateral wall. As for the medial wall, it was the anteromedial wall that undertook the pivotal stabilizing responsibility compared with the counterpart of the posteromedial wall, which indicated that orthopedic surgeons should attach great importance to the anteromedial wall and restore its cortex support in an operating theatre.

## Data Availability

The original contributions presented in the study are included in the article/Supplementary Material, further inquiries can be directed to the corresponding authors.

## Abbreviations

AO/OTA, Arbeitsgemeinschaft fur Osteosynthesefragen classification; PFNA, proximal femoral nail anti-rotation; InterTAN, intertrochanteric antegrade nail; CT, computed tomography; DEXA, dual-energy x-ray absorptiometry; AM, anterior medial wall; PM, posterior medial wall; AL, anterior lateral wall; PL, posterior lateral wall; PML, posterior coronal bone defect model; IM, intramedullary nail; TAD, tip-apex distance; FE, finite element models; VMS, von Mises stress.
